# The complete chloroplast genome and phylogenetic analysis of *Sida szechuensis* matsuda (Malvaceae)

**DOI:** 10.1080/23802359.2021.1987161

**Published:** 2021-10-07

**Authors:** Dong-Qin Guo, Hai-Ling Li, Chang Liu, Hua Zhang, Hui-Hui Du, Nong Zhou

**Affiliations:** a College of Biology and Food Engineering, Chongqing Three Gorges University, Chongqing, China; b Engineering Laboratory of Chongqing for Green Planting and Deep Processing of Genuine Medicinal Materials in Three Gorges Reservoir Area, Chongqing Three Gorges University, Chongqing, China; c Nanjing Institute for Comprehensive Utilization of Wild Plants, Nanjing, China

**Keywords:** *Sida szechuensis*, Malvaceae, complete chloroplast genome, phylogenetic analysis

## Abstract

*Sida szechuensis* Matsuda is an economically and medicinally important plant. Here, we report the first chloroplast (cp) genome of the genus *Sida* (*S. szechuensis*). The complete cp genome is 159,878 bp in length with an overall GC content of 36.9% and consists of a large single copy region (LSC, 89,426 bp), a small single copy region (SSC, 114,715 bp), and a pair of inverted repeat regions (IRa and IRb, 25,288 bp). The genome encodes 111 unique genes, including 78 protein-coding genes, 29 tRNA genes, 4 rRNA genes, and 1 pseudogene. Phylogenetic analysis constructed using the maximum likelihood (ML) method showed that *Sida* was closely related to *Malvastrum* and *Malva*.

*Sida szechuensis* Matsuda 1918 belongs to the family Malvaceae, genus *Sida*. There are 100 genera in the Malvaceae, between 100 and 150 species in the *Sida* worldwide, and 14 species (six endemic) of *Sida* in China (Editorial Committee of Flora of China 2007). *Sida szechuensis* is a medicinal plant that grows in the highland approximately one to two thousand meters above sea level in Southwest China and is useful for treating ulcers, furuncle, dysentery, and blood stasis in ethnopharmacology (Xiao et al. 1997). Previous studies on *S. szechuensis* have focused on its chemical composition and medicinal value. However, analysis based on its complete cp genome is still lacking. In this study, the cp genome of *S. szechuensis* was obtained by next-generation sequencing technology. We analyzed the characteristics of the cp genome of *S. szechuensis* and revealed its phylogenetic relationships with other species in Malvaceae. These contents enrich the study of the cp genome information of the genus *Sida* and are important for further studies that evaluate the germplasm and molecular phylogeny of Malvaceae.

In this study, young leaf samples of *S. szechuensis* were collected from Dali, Yunnan Province, China (100°22′31.44″E, 25°45′5.45″N). The specimen was deposited at the Herbarium of Medicinal Plants and Crude Drugs of the College of Pharmacy, Dali University (De-Quan Zhang, zhangdeq2008@126.com) under the voucher number ZSY110.

Total genomic DNA was extracted using the modified CTAB method from the dry and healthy leaves (Doyle 1987; Yang et al. 2014). Whole-genome sequencing was obtained via the Illumina HiSeq 2500 (Novogene, Tianjin, China) platform with the paired-end (2 × 300 bp) library. After removing the adapters and low-quality reads, the high-quality reads were assembled to the cp genome by GetOrganelle with the parameters 21, 45, 65, 85, and 105. (Jin et al. 2020). Then the assembled cp genome of *S. szechuensis* was annotated using Geneious 11.0.4 (Kearse et al. 2012) with the sequence of *Malvastrum coromandelianum* (MK860037) as the reference, and the stop codons of the *ndhF*, *ccsA* and *ndhl* genes were corrected manually. Finally, the cp genome sequences were deposited in GenBank (accession numbers, MT773597).

The cp genome of *S. szechuensis* has a total length of 159,878 bp and presents a typical quadripartite structure. It contains a pair of inverted repeat regions (IR, 25,288 bp) that were separated by a large single copy region (LSC, 89,426 bp) and a small single copy region (SSC, 114,715 bp). The cp genome comprised a total of 111 genes, including 78 protein-coding genes, 29 rRNA genes, 4 tRNA genes and 1 pseudogene (*infA*). The overall GC content of *S. szechuensis* was 36.9%, while those of the IR regions (42.9%) were higher than those of LSC (34.7%) and SSC (31.5%) regions. However, previous reports showed that the GC content varies in different regions of chloroplast genomes, but the IR regions have a high GC content due to the presence of rRNAs, which contain a high GC content (Mehmood, Abdullah Ubaid, Bao, et al. 2020; Mehmood, Abdullah Ubaid, Shahzadi, et al. 2020; Mehmood, Abdullah Shahzadi, et al. 2020).

We downloaded 19 complete cp genome sequences from GenBank. Thirteen sequences of Malvaceae, four sequences of the genera *Heritiera* and *Sterculia* from the family Sterculiaceae and two sequences of *Elaeocarpus braceanus* and *Elaeocarpus japonicus* from the family Elaeocarpaceae were used as outgroups. All sequences were initially aligned using MAFFT V.7.149 (Katoh and Standley 2013). Then, the maximum likelihood tree was generated by RAxML (Stamatakis 2014) with 1000 bootstrap replicates under the GTRGAMMAI substitution model. The results of the phylogenetic analysis strongly supported that *Sida* is closely related to *Malvastrum* and *Malva* (Figure 1). These results will provide valuable information for phylogenetic and evolutionary studies on Malvaceae.

**Figure 1.**
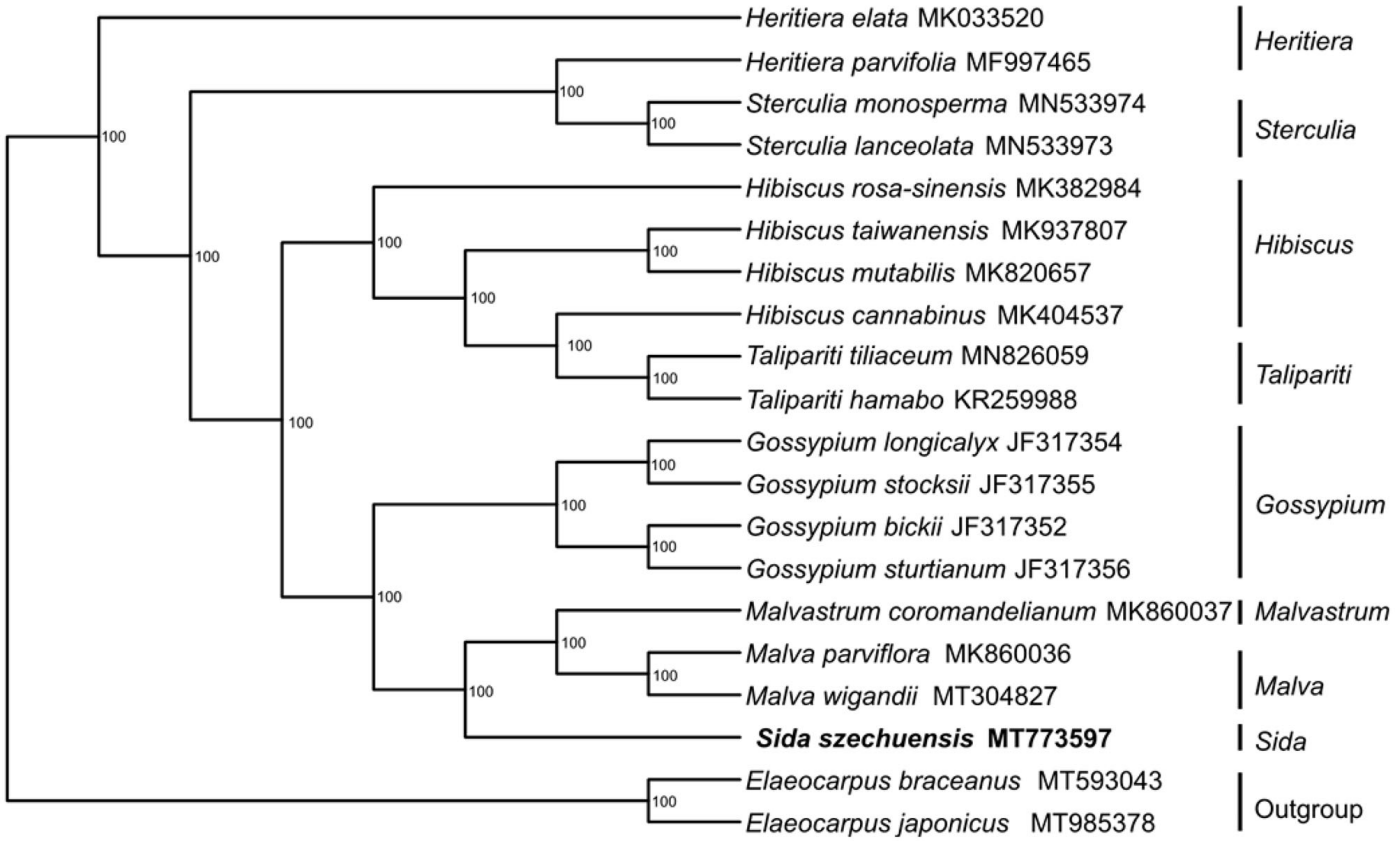
Maximum-likelihood tree showing the phylogenetic position of *Sida szechuensis* based on the complete chloroplast genome sequences of 20 species. Bootstrap support values (1000 replicates) are shown next to the nodes.

## Data Availability

The genome sequence data that support the findings of this study are openly available in GenBank of NCBI under accession no. MT773597 (https://www.ncbi.nlm.nih.gov/nuccore/MT773597). The associated BioProject, SRA, and BioSample numbers are PRJNA746696, SRR15145519, and SAMN20236522, respectively.
